# Tris(2-formyl­phenolato-κ^2^ *O*,*O*′)(1,10-phenanthroline-κ^2^ *N*,*N*′)samarium(III)

**DOI:** 10.1107/S1600536813016139

**Published:** 2013-07-03

**Authors:** Yun Zhong, Jinbing Yang, Ling Hu

**Affiliations:** a School of Basical Science, East China Jiaotong University, Nanchang 330013, People’s Republic of China

## Abstract

In the title compound, [Sm(C_7_H_5_O_2_)_3_(C_12_H_8_N_2_)], the Sm^III^ cation is coordinated by six O atoms from three bidentate 2-formyl­phenolate ligands and by two N atoms from 1,10-phenanthroline ligand. The resulting SmN_2_O6 coordination polyhedron is a distorted square anti­prism. In the crystal, C—H⋯O inter­actions connect mol­ecules into chains along the *b*-axis direction. In addition, π–π stacking inter­actions are observed with centroid–centroid distances in the range 3.6422 (13)–3.7329 (13) Å.

## Related literature

For the structures of metal complexes with 2-formyl­phenolate ligands, see: Li & Chen (2006 ▶); Li *et al.* (2007 ▶); Xiao & Zhang (2008 ▶); Yang *et al.* (2007 ▶).
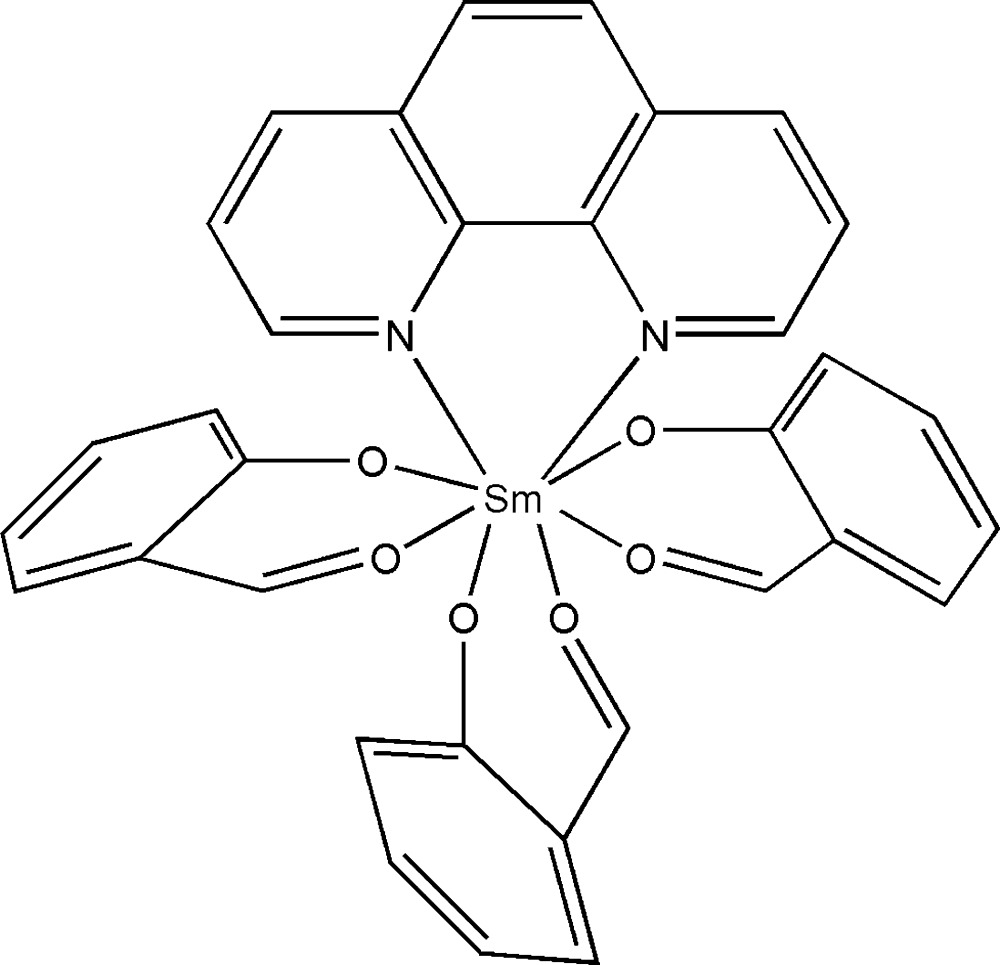


## Experimental

### 

#### Crystal data

[Sm(C_7_H_5_O_2_)_3_(C_12_H_8_N_2_)]
*M* *_r_* = 693.88
Monoclinic, 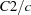

*a* = 24.1921 (9) Å
*b* = 14.6474 (6) Å
*c* = 17.4681 (7) Å
β = 115.28°
*V* = 5596.8 (4) Å^3^
*Z* = 8
Mo *K*α radiation
μ = 2.15 mm^−1^
*T* = 298 K
0.35 × 0.30 × 0.20 mm

#### Data collection

Bruker SMART CCD area-detector diffractometer
Absorption correction: multi-scan (*SADABS*; Sheldrick, 1996 ▶) *T*_min_ = 0.520, *T*_max_ = 0.673
20817 measured reflections
5066 independent reflections
4612 reflections with *I* > 2σ(*I*)
*R*_int_ = 0.017

#### Refinement

*R*[*F* ^2^ > 2σ(*F* ^2^)] = 0.017
*wR*(*F* ^2^) = 0.049
*S* = 1.09
5066 reflections
379 parameters
H-atom parameters constrained
Δρ_max_ = 0.44 e Å^−3^
Δρ_min_ = −0.32 e Å^−3^

### 

Data collection: *SMART* (Bruker, 1998 ▶); cell refinement: *SAINT* (Bruker, 1999 ▶); data reduction: *SAINT*; program(s) used to solve structure: *SHELXS97* (Sheldrick, 2008 ▶); program(s) used to refine structure: *SHELXL97* (Sheldrick, 2008 ▶); molecular graphics: *SHELXTL* (Sheldrick, 2008 ▶); software used to prepare material for publication: *SHELXL97*.

## Supplementary Material

Crystal structure: contains datablock(s) I, global. DOI: 10.1107/S1600536813016139/ff2109sup1.cif

Structure factors: contains datablock(s) I. DOI: 10.1107/S1600536813016139/ff2109Isup2.hkl

Additional supplementary materials:  crystallographic information; 3D view; checkCIF report

## Figures and Tables

**Table 1:** Selected bond lengths (Å)

N1—Sm1	2.6398 (18)
N2—Sm1	2.602 (2)
O1—Sm1	2.2906 (16)
O2—Sm1	2.4977 (16)
O3—Sm1	2.2925 (15)
O4—Sm1	2.4714 (18)
O5—Sm1	2.3025 (15)
O6—Sm1	2.4888 (16)

**Table 2:** Hydrogen-bond geometry (Å, °)

*D*—H⋯*A*	*D*—H	H⋯*A*	*D*⋯*A*	*D*—H⋯*A*
C19—H19⋯O6^i^	0.93	2.46	3.326 (3)	154

Symmetry code: (i) 
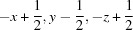
.

**Fig. 1.**
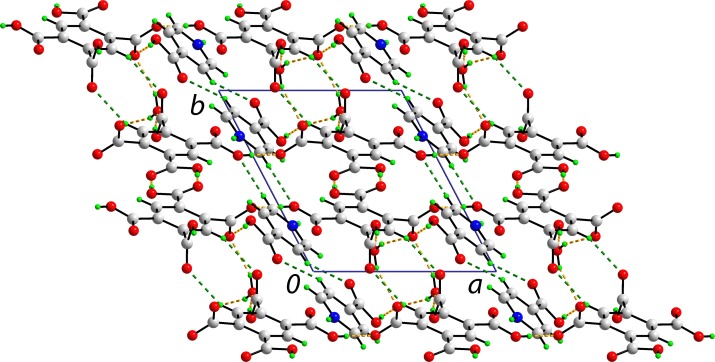
The molecular structure of the title compound with 50% probability displacement ellipsoids for non-H atoms.

## supplementary crystallographic information

### Comment

Many metal complexes with Schiff base ligands, which had been synthesized by salicylaldehyde and amine, have been reported. However, only a few metal complexes with 2-formylphenolate ligands had been reported so far. In metal complexes with 2-formylphenolate ligands, the metal ion had been likely coordinated by O atoms from bidentate 2-formylphenolate ligands in a square-planar geometry.(Li *et al.*,2006; Yang *et al.*, 2007; Xiao & Zhang, 2008; Li *et al.*, 2007)

The title compound, [Sm(C_7_H_5_O_2_)_3_(C_12_H_8_N_2_)], was obtained by the reaction of salicylaldehyde, 1,10-phenanthroline and samarium(III) chloride in methanol. The Sm^III^ cation is coordinated by six O atoms from three bidentate 2-formylphenolate ligands and by two N atoms from 1,10-phenanthroline ligand. The Sm—O distances range from 2.2906 (16) to 2.4977 (16) Å, and the Sm—N distances range from 2.602 (2) to 2.6398 (18) Å.

### Experimental

SmCl_3 _(0.26 g, 1.0 mmol), salicylaldehyde (0.37 g, 3.0 mmol) and triethylamine (0.3 g, 3 mmol) was stirred for 1 h in methanol (20 ml) at reflux temperature and cooled to room temperature. To this solution, a methanol solution (5 ml) containing 1,10-phenanthroline (0.18 g, 1 mmol) was added. The mixture was stirred for 1 h at room temperature and the precipitate was filtered off. Brown crystals were obtained by a slow evaporation of the filtrate.

### Refinement

H atoms were placed in calculated positions with C—H = 0.97 and O—H = 0.82 Å, and refined in riding mode with *U*_iso_(H) = 1.2*U*_eq_(C) and 1.5*U*_eq_(O). The highest peak in the final difference Fourier map is 0.93 Å apart from the Sm1 atom.

### Crystal data

**Table:**

[Sm(C_7_H_5_O_2_)_3_(C_12_H_8_N_2_)]	*F*(000) = 2760
*M**_r_* = 693.88	*D*_x_ = 1.647 Mg m^−^^3^
Monoclinic, *C*2/*c*	Mo *K*α radiation, λ = 0.71073 Å
Hall symbol: -C 2yc	Cell parameters from 9986 reflections
*a* = 24.1921 (9) Å	θ = 2.3–28.4°
*b* = 14.6474 (6) Å	µ = 2.15 mm^−^^1^
*c* = 17.4681 (7) Å	*T* = 298 K
β = 115.28°	Prism, brown
*V* = 5596.8 (4) Å^3^	0.35 × 0.30 × 0.20 mm
*Z* = 8	

### Data collection

**Table:**

Bruker SMART CCD area-detector diffractometer	5066 independent reflections
Radiation source: fine-focus sealed tube	4612 reflections with *I* > 2σ(*I*)
Graphite monochromator	*R*_int_ = 0.017
phi and ω scans	θ_max_ = 25.3°, θ_min_ = 2.3°
Absorption correction: multi-scan (*SADABS*; Sheldrick, 1996)	*h* = −28→28
*T*_min_ = 0.520, *T*_max_ = 0.673	*k* = −17→17
20817 measured reflections	*l* = −20→20

### Refinement

**Table:**

Refinement on *F*^2^	Primary atom site location: structure-invariant direct methods
Least-squares matrix: full	Secondary atom site location: difference Fourier map
*R*[*F*^2^ > 2σ(*F*^2^)] = 0.017	Hydrogen site location: inferred from neighbouring sites
*wR*(*F*^2^) = 0.049	H-atom parameters constrained
*S* = 1.09	*w* = 1/[σ^2^(*F*_o_^2^) + (0.0247*P*)^2^ + 4.8252*P*] where *P* = (*F*_o_^2^ + 2*F*_c_^2^)/3
5066 reflections	(Δ/σ)_max_ = 0.001
379 parameters	Δρ_max_ = 0.44 e Å^−^^3^
0 restraints	Δρ_min_ = −0.32 e Å^−^^3^

### Special details

**Table:**

Geometry. All e.s.d.'s (except the e.s.d. in the dihedral angle between two l.s. planes) are estimated using the full covariance matrix. The cell e.s.d.'s are taken into account individually in the estimation of e.s.d.'s in distances, angles and torsion angles; correlations between e.s.d.'s in cell parameters are only used when they are defined by crystal symmetry. An approximate (isotropic) treatment of cell e.s.d.'s is used for estimating e.s.d.'s involving l.s. planes.
Refinement. Refinement of *F*^2^ against ALL reflections. The weighted *R*-factor *wR* and goodness of fit *S* are based on *F*^2^, conventional *R*-factors *R* are based on *F*, with *F* set to zero for negative *F*^2^. The threshold expression of *F*^2^ > σ(*F*^2^) is used only for calculating *R*-factors(gt) *etc*. and is not relevant to the choice of reflections for refinement. *R*-factors based on *F*^2^ are statistically about twice as large as those based on *F*, and *R*- factors based on ALL data will be even larger.

### Fractional atomic coordinates and isotropic or equivalent isotropic displacement parameters (Å^2^)

**Table:**

	*x*	*y*	*z*	*U*_iso_*/*U*_eq_	
C1	0.31169 (10)	0.86165 (16)	0.37537 (16)	0.0465 (5)	
H1	0.3199	0.8625	0.3280	0.056*	
C2	0.35943 (11)	0.84125 (17)	0.45406 (17)	0.0528 (6)	
H2	0.3985	0.8294	0.4585	0.063*	
C3	0.34823 (11)	0.83896 (16)	0.52364 (16)	0.0518 (6)	
H3	0.3796	0.8258	0.5764	0.062*	
C4	0.28917 (11)	0.85648 (14)	0.51595 (14)	0.0433 (5)	
C5	0.27276 (13)	0.85179 (16)	0.58590 (15)	0.0536 (6)	
H5	0.3027	0.8387	0.6398	0.064*	
C6	0.21547 (13)	0.86576 (17)	0.57504 (16)	0.0560 (6)	
H6	0.2062	0.8621	0.6214	0.067*	
C7	0.16799 (12)	0.88631 (16)	0.49350 (16)	0.0485 (6)	
C8	0.10657 (14)	0.89827 (19)	0.4777 (2)	0.0629 (7)	
H8	0.0953	0.8947	0.5222	0.075*	
C9	0.06353 (14)	0.9149 (2)	0.3984 (2)	0.0679 (8)	
H9	0.0227	0.9222	0.3878	0.081*	
C10	0.08197 (13)	0.9208 (2)	0.3334 (2)	0.0635 (8)	
H10	0.0524	0.9330	0.2790	0.076*	
C11	0.18228 (10)	0.89213 (14)	0.42354 (15)	0.0395 (5)	
C12	0.24393 (10)	0.87700 (13)	0.43492 (13)	0.0365 (5)	
C13	0.08129 (10)	0.71970 (16)	0.16434 (16)	0.0470 (5)	
C14	0.02349 (12)	0.68030 (19)	0.1451 (2)	0.0747 (9)	
H14	−0.0062	0.7149	0.1522	0.090*	
C15	0.01027 (15)	0.59231 (19)	0.1162 (3)	0.0831 (11)	
H15	−0.0285	0.5691	0.1032	0.100*	
C16	0.05290 (14)	0.53738 (19)	0.1060 (2)	0.0723 (9)	
H16	0.0429	0.4782	0.0853	0.087*	
C17	0.10973 (14)	0.57144 (17)	0.12672 (18)	0.0596 (7)	
H17	0.1390	0.5344	0.1210	0.071*	
C18	0.12548 (10)	0.66124 (15)	0.15659 (14)	0.0424 (5)	
C19	0.18756 (11)	0.68770 (16)	0.18207 (16)	0.0486 (6)	
H19	0.2133	0.6433	0.1770	0.058*	
C20	0.27639 (11)	0.93203 (15)	0.14703 (15)	0.0424 (5)	
C21	0.33951 (11)	0.94760 (18)	0.17270 (17)	0.0511 (6)	
H21	0.3624	0.9743	0.2251	0.061*	
C22	0.36752 (14)	0.92430 (19)	0.1224 (2)	0.0651 (8)	
H22	0.4093	0.9343	0.1419	0.078*	
C23	0.33551 (17)	0.8860 (3)	0.0428 (2)	0.0798 (10)	
H23	0.3554	0.8703	0.0094	0.096*	
C24	0.27492 (16)	0.8721 (3)	0.0150 (2)	0.0764 (9)	
H24	0.2530	0.8480	−0.0388	0.092*	
C25	0.24352 (13)	0.89319 (18)	0.06522 (16)	0.0534 (6)	
C26	0.17925 (14)	0.8767 (2)	0.03053 (17)	0.0634 (7)	
H26	0.1611	0.8570	−0.0254	0.076*	
C27	0.06299 (10)	1.08986 (14)	0.14602 (15)	0.0391 (5)	
C28	0.00641 (11)	1.11852 (17)	0.08153 (17)	0.0509 (6)	
H28	−0.0168	1.0774	0.0395	0.061*	
C29	−0.01491 (13)	1.20557 (19)	0.0797 (2)	0.0681 (8)	
H29	−0.0522	1.2224	0.0363	0.082*	
C30	0.01791 (14)	1.2692 (2)	0.1412 (3)	0.0832 (10)	
H30	0.0025	1.3276	0.1400	0.100*	
C31	0.07337 (14)	1.24430 (19)	0.2038 (2)	0.0715 (8)	
H31	0.0958	1.2868	0.2449	0.086*	
C32	0.09729 (10)	1.15610 (16)	0.20742 (15)	0.0457 (5)	
C33	0.15714 (11)	1.13771 (18)	0.27111 (16)	0.0525 (6)	
H33	0.1758	1.1851	0.3088	0.063*	
N1	0.25528 (8)	0.87989 (12)	0.36524 (11)	0.0394 (4)	
N2	0.13901 (9)	0.91009 (13)	0.34420 (13)	0.0469 (5)	
O1	0.09273 (7)	0.80347 (11)	0.18874 (13)	0.0598 (5)	
O2	0.21128 (7)	0.76192 (11)	0.20986 (11)	0.0520 (4)	
O3	0.25049 (7)	0.95453 (11)	0.19573 (9)	0.0441 (4)	
O4	0.14503 (8)	0.88545 (14)	0.06560 (11)	0.0590 (4)	
O5	0.08275 (7)	1.00738 (10)	0.14640 (10)	0.0466 (4)	
O6	0.18739 (8)	1.06706 (11)	0.28260 (11)	0.0519 (4)	
Sm1	0.165805 (5)	0.913618 (7)	0.214933 (7)	0.03584 (5)	

### Atomic displacement parameters (Å^2^)

**Table:**

	*U*^11^	*U*^22^	*U*^33^	*U*^12^	*U*^13^	*U*^23^
C1	0.0328 (12)	0.0541 (14)	0.0520 (14)	0.0006 (10)	0.0175 (10)	0.0100 (11)
C2	0.0300 (12)	0.0578 (14)	0.0624 (16)	−0.0004 (10)	0.0120 (11)	0.0116 (12)
C3	0.0409 (13)	0.0494 (13)	0.0483 (14)	−0.0062 (10)	0.0030 (11)	0.0079 (11)
C4	0.0469 (13)	0.0336 (11)	0.0414 (12)	−0.0087 (9)	0.0112 (10)	−0.0009 (9)
C5	0.0689 (18)	0.0470 (13)	0.0371 (13)	−0.0123 (12)	0.0152 (12)	−0.0046 (10)
C6	0.0786 (19)	0.0516 (14)	0.0466 (14)	−0.0115 (13)	0.0353 (14)	−0.0101 (11)
C7	0.0618 (16)	0.0388 (11)	0.0531 (14)	−0.0061 (11)	0.0322 (13)	−0.0078 (10)
C8	0.0698 (19)	0.0687 (17)	0.0714 (19)	−0.0014 (14)	0.0505 (17)	−0.0100 (14)
C9	0.0515 (17)	0.081 (2)	0.087 (2)	0.0063 (14)	0.0441 (17)	−0.0033 (16)
C10	0.0419 (15)	0.088 (2)	0.0653 (18)	0.0100 (13)	0.0270 (14)	0.0082 (14)
C11	0.0443 (13)	0.0318 (10)	0.0471 (13)	−0.0019 (9)	0.0239 (11)	−0.0010 (9)
C12	0.0380 (11)	0.0287 (10)	0.0408 (12)	−0.0051 (8)	0.0151 (9)	0.0001 (8)
C13	0.0374 (12)	0.0413 (12)	0.0581 (14)	−0.0017 (10)	0.0164 (11)	0.0016 (10)
C14	0.0397 (14)	0.0526 (15)	0.126 (3)	−0.0060 (12)	0.0298 (16)	−0.0042 (17)
C15	0.0470 (17)	0.0553 (18)	0.121 (3)	−0.0153 (13)	0.0111 (18)	0.0009 (17)
C16	0.0662 (19)	0.0404 (14)	0.084 (2)	−0.0048 (13)	0.0068 (16)	−0.0043 (13)
C17	0.0625 (17)	0.0431 (13)	0.0621 (17)	0.0061 (12)	0.0162 (14)	−0.0036 (11)
C18	0.0422 (12)	0.0395 (11)	0.0407 (12)	0.0028 (9)	0.0131 (10)	0.0030 (9)
C19	0.0441 (13)	0.0457 (13)	0.0558 (14)	0.0096 (11)	0.0212 (11)	0.0013 (11)
C20	0.0467 (13)	0.0407 (11)	0.0424 (12)	0.0048 (10)	0.0214 (11)	0.0114 (9)
C21	0.0454 (14)	0.0561 (14)	0.0552 (15)	0.0038 (11)	0.0246 (12)	0.0108 (12)
C22	0.0542 (17)	0.0742 (19)	0.079 (2)	0.0143 (13)	0.0403 (16)	0.0225 (15)
C23	0.083 (2)	0.103 (2)	0.077 (2)	0.024 (2)	0.056 (2)	0.0081 (19)
C24	0.081 (2)	0.102 (2)	0.0538 (17)	0.0125 (19)	0.0354 (16)	−0.0070 (16)
C25	0.0563 (16)	0.0635 (15)	0.0414 (13)	0.0075 (12)	0.0220 (12)	0.0021 (11)
C26	0.0644 (18)	0.0795 (19)	0.0384 (14)	0.0023 (15)	0.0143 (13)	−0.0093 (13)
C27	0.0354 (11)	0.0417 (12)	0.0431 (12)	0.0006 (9)	0.0195 (10)	0.0004 (9)
C28	0.0374 (12)	0.0518 (13)	0.0578 (15)	0.0042 (11)	0.0149 (11)	0.0007 (11)
C29	0.0426 (15)	0.0593 (17)	0.091 (2)	0.0128 (12)	0.0182 (15)	0.0096 (15)
C30	0.0615 (19)	0.0500 (16)	0.130 (3)	0.0175 (14)	0.034 (2)	−0.0042 (18)
C31	0.0613 (18)	0.0531 (16)	0.097 (2)	−0.0012 (13)	0.0307 (17)	−0.0223 (15)
C32	0.0397 (12)	0.0462 (12)	0.0526 (14)	−0.0019 (10)	0.0211 (11)	−0.0069 (10)
C33	0.0495 (14)	0.0513 (14)	0.0524 (14)	−0.0113 (12)	0.0179 (12)	−0.0159 (11)
N1	0.0321 (9)	0.0423 (9)	0.0424 (10)	−0.0011 (8)	0.0145 (8)	0.0068 (8)
N2	0.0340 (10)	0.0582 (12)	0.0500 (12)	0.0033 (8)	0.0193 (9)	0.0071 (9)
O1	0.0401 (9)	0.0434 (9)	0.1014 (15)	−0.0057 (7)	0.0356 (10)	−0.0149 (9)
O2	0.0372 (9)	0.0495 (10)	0.0678 (11)	0.0019 (7)	0.0211 (8)	−0.0022 (8)
O3	0.0427 (9)	0.0518 (9)	0.0408 (8)	−0.0062 (7)	0.0207 (7)	−0.0004 (7)
O4	0.0445 (10)	0.0777 (12)	0.0443 (10)	−0.0014 (9)	0.0089 (8)	−0.0070 (9)
O5	0.0371 (8)	0.0414 (8)	0.0482 (9)	0.0040 (7)	0.0056 (7)	−0.0046 (7)
O6	0.0391 (9)	0.0502 (9)	0.0532 (10)	−0.0047 (7)	0.0071 (8)	−0.0056 (8)
Sm1	0.02721 (7)	0.03827 (7)	0.03935 (8)	−0.00068 (4)	0.01164 (5)	0.00116 (4)

### Geometric parameters (Å, º)

**Table:**

C1—N1	1.326 (3)	C19—H19	0.9300
C1—C2	1.399 (3)	C20—O3	1.296 (3)
C1—H1	0.9300	C20—C21	1.414 (3)
C2—C3	1.354 (4)	C20—C25	1.424 (4)
C2—H2	0.9300	C21—C22	1.363 (4)
C3—C4	1.400 (3)	C21—H21	0.9300
C3—H3	0.9300	C22—C23	1.387 (5)
C4—C12	1.403 (3)	C22—H22	0.9300
C4—C5	1.439 (3)	C23—C24	1.348 (5)
C5—C6	1.332 (4)	C23—H23	0.9300
C5—H5	0.9300	C24—C25	1.418 (4)
C6—C7	1.429 (4)	C24—H24	0.9300
C6—H6	0.9300	C25—C26	1.428 (4)
C7—C8	1.401 (4)	C26—O4	1.229 (3)
C7—C11	1.407 (3)	C26—H26	0.9300
C8—C9	1.352 (5)	C27—O5	1.298 (3)
C8—H8	0.9300	C27—C28	1.415 (3)
C9—C10	1.390 (4)	C27—C32	1.421 (3)
C9—H9	0.9300	C28—C29	1.371 (4)
C10—N2	1.320 (3)	C28—H28	0.9300
C10—H10	0.9300	C29—C30	1.387 (4)
C11—N2	1.359 (3)	C29—H29	0.9300
C11—C12	1.435 (3)	C30—C31	1.369 (4)
C12—N1	1.358 (3)	C30—H30	0.9300
C13—O1	1.290 (3)	C31—C32	1.406 (4)
C13—C14	1.414 (3)	C31—H31	0.9300
C13—C18	1.421 (3)	C32—C33	1.425 (3)
C14—C15	1.371 (4)	C33—O6	1.233 (3)
C14—H14	0.9300	C33—H33	0.9300
C15—C16	1.379 (5)	N1—Sm1	2.6398 (18)
C15—H15	0.9300	N2—Sm1	2.602 (2)
C16—C17	1.358 (4)	O1—Sm1	2.2906 (16)
C16—H16	0.9300	O2—Sm1	2.4977 (16)
C17—C18	1.406 (3)	O3—Sm1	2.2925 (15)
C17—H17	0.9300	O4—Sm1	2.4714 (18)
C18—C19	1.426 (3)	O5—Sm1	2.3025 (15)
C19—O2	1.228 (3)	O6—Sm1	2.4888 (16)
			
N1—C1—C2	123.0 (2)	C23—C24—H24	118.9
N1—C1—H1	118.5	C25—C24—H24	118.9
C2—C1—H1	118.5	C24—C25—C20	119.4 (3)
C3—C2—C1	119.3 (2)	C24—C25—C26	118.4 (3)
C3—C2—H2	120.3	C20—C25—C26	122.2 (2)
C1—C2—H2	120.3	O4—C26—C25	127.9 (2)
C2—C3—C4	119.8 (2)	O4—C26—H26	116.0
C2—C3—H3	120.1	C25—C26—H26	116.0
C4—C3—H3	120.1	O5—C27—C28	120.6 (2)
C3—C4—C12	117.4 (2)	O5—C27—C32	122.4 (2)
C3—C4—C5	123.4 (2)	C28—C27—C32	116.9 (2)
C12—C4—C5	119.2 (2)	C29—C28—C27	121.4 (2)
C6—C5—C4	121.3 (2)	C29—C28—H28	119.3
C6—C5—H5	119.3	C27—C28—H28	119.3
C4—C5—H5	119.3	C28—C29—C30	121.4 (3)
C5—C6—C7	121.3 (2)	C28—C29—H29	119.3
C5—C6—H6	119.4	C30—C29—H29	119.3
C7—C6—H6	119.4	C31—C30—C29	118.8 (3)
C8—C7—C11	117.0 (2)	C31—C30—H30	120.6
C8—C7—C6	123.8 (2)	C29—C30—H30	120.6
C11—C7—C6	119.2 (2)	C30—C31—C32	121.6 (3)
C9—C8—C7	120.8 (3)	C30—C31—H31	119.2
C9—C8—H8	119.6	C32—C31—H31	119.2
C7—C8—H8	119.6	C31—C32—C27	119.8 (2)
C8—C9—C10	118.2 (3)	C31—C32—C33	118.0 (2)
C8—C9—H9	120.9	C27—C32—C33	122.1 (2)
C10—C9—H9	120.9	O6—C33—C32	128.3 (2)
N2—C10—C9	123.8 (3)	O6—C33—H33	115.8
N2—C10—H10	118.1	C32—C33—H33	115.8
C9—C10—H10	118.1	C1—N1—C12	117.69 (19)
N2—C11—C7	122.0 (2)	C1—N1—Sm1	122.02 (15)
N2—C11—C12	118.3 (2)	C12—N1—Sm1	120.23 (13)
C7—C11—C12	119.8 (2)	C10—N2—C11	118.2 (2)
N1—C12—C4	122.8 (2)	C10—N2—Sm1	120.39 (18)
N1—C12—C11	117.84 (19)	C11—N2—Sm1	121.33 (15)
C4—C12—C11	119.3 (2)	C13—O1—Sm1	140.77 (15)
O1—C13—C14	121.2 (2)	C19—O2—Sm1	131.52 (15)
O1—C13—C18	122.7 (2)	C20—O3—Sm1	137.92 (15)
C14—C13—C18	116.1 (2)	C26—O4—Sm1	131.83 (17)
C15—C14—C13	121.5 (3)	C27—O5—Sm1	142.69 (14)
C15—C14—H14	119.2	C33—O6—Sm1	133.70 (15)
C13—C14—H14	119.2	O1—Sm1—O3	144.74 (6)
C14—C15—C16	121.8 (3)	O1—Sm1—O5	83.60 (6)
C14—C15—H15	119.1	O3—Sm1—O5	113.82 (6)
C16—C15—H15	119.1	O1—Sm1—O4	83.10 (7)
C17—C16—C15	118.6 (3)	O3—Sm1—O4	71.39 (6)
C17—C16—H16	120.7	O5—Sm1—O4	79.16 (6)
C15—C16—H16	120.7	O1—Sm1—O6	135.93 (6)
C16—C17—C18	121.7 (3)	O3—Sm1—O6	79.29 (6)
C16—C17—H17	119.1	O5—Sm1—O6	70.59 (5)
C18—C17—H17	119.1	O4—Sm1—O6	123.98 (6)
C17—C18—C13	120.2 (2)	O1—Sm1—O2	71.36 (5)
C17—C18—C19	116.9 (2)	O3—Sm1—O2	78.04 (6)
C13—C18—C19	122.8 (2)	O5—Sm1—O2	144.28 (5)
O2—C19—C18	128.0 (2)	O4—Sm1—O2	73.03 (6)
O2—C19—H19	116.0	O6—Sm1—O2	144.49 (5)
C18—C19—H19	116.0	O1—Sm1—N2	72.68 (7)
O3—C20—C21	120.7 (2)	O3—Sm1—N2	134.55 (6)
O3—C20—C25	122.8 (2)	O5—Sm1—N2	88.71 (6)
C21—C20—C25	116.6 (2)	O4—Sm1—N2	154.01 (6)
C22—C21—C20	121.5 (3)	O6—Sm1—N2	71.66 (6)
C22—C21—H21	119.3	O2—Sm1—N2	106.85 (6)
C20—C21—H21	119.3	O1—Sm1—N1	107.57 (6)
C21—C22—C23	121.9 (3)	O3—Sm1—N1	77.88 (5)
C21—C22—H22	119.1	O5—Sm1—N1	142.33 (6)
C23—C22—H22	119.1	O4—Sm1—N1	136.83 (6)
C24—C23—C22	118.5 (3)	O6—Sm1—N1	77.30 (6)
C24—C23—H23	120.7	O2—Sm1—N1	71.48 (6)
C22—C23—H23	120.7	N2—Sm1—N1	62.33 (6)
C23—C24—C25	122.1 (3)		

### Hydrogen-bond geometry (Å, º)

**Table:**

*D*—H···*A*	*D*—H	H···*A*	*D*···*A*	*D*—H···*A*
C19—H19···O6^i^	0.93	2.46	3.326 (3)	154

Symmetry code: (i) −*x*+1/2, *y*−1/2, −*z*+1/2.
